# Immunomodulatory Activity and Phytochemical Profile of Infusions from Cleavers Herb

**DOI:** 10.3390/molecules25163721

**Published:** 2020-08-14

**Authors:** Tetiana Ilina, Weronika Skowrońska, Natalia Kashpur, Sebastian Granica, Agnieszka Bazylko, Alla Kovalyova, Olga Goryacha, Oleh Koshovyi

**Affiliations:** 1 National University of Pharmacy, 53-Pushkinska str, 61002 Kharkiv, Ukraine; ilyinatany86@gmail.com (T.I.); allapharm@yahoo.com (A.K.); helgagnosy@gmail.com (O.G.); oleh.koshovyi@gmail.com (O.K.); 2 Department of Pharmacognosy and Molecular Basis of Phytoteraphy, Medical University of Warsaw, 1 Banacha str, 02-097 Warsaw, Poland; weronika.skowronska@wum.edu.pl (W.S.); agnieszka.bazylko@wum.edu.pl (A.B.); 3 Mechnikov Institute of Microbiology and Immunology, National Academy of Medical Sciences of Ukraine, 14/16-Pushkinska str., 61057 Kharkiv, Ukraine; natali.kashpur@ukr.net

**Keywords:** *Galium aparine*, cleavers, antioxidant activity, immunomodulatory activity, polyphenols, skin

## Abstract

Extracts from aerial parts of *G. aparine* (cleavers) constitute a herbal remedy with monography in British Herbal Pharmacopeia. On the European market, there are several drugs and food supplements consisting of *Galium* extracts. In folk medicine, cleavers was used topically in Europe, Asia, and the Americas to treat skin diseases. In several remedies, cleavers is also listed as an immunomodulatory active herb influencing the defense response of the human body. The aim of this study was to investigate the immunostimulatory activity and antioxidant potential in vitro of a raw infusion of cleavers and bioactive fractions. The functional activity of lymphocytes in the reaction of the lymphocyte blast transformation (RLBT) method was used for immunomodulatory activity assays and direct scavenging of 2,2-diphenyl-1-picrylhydrazyl (DPPH), nitric oxide (NO), and hydrogen peroxide (H_2_O_2_) was chosen for the examination of antioxidant activity. It was shown that both the raw extract and fractions show significant immunostimulatory and scavenging activities. The obtained data partially justify the traditional use of cleavers as topical remedy for skin infections and for wounds.

## 1. Introduction

*Galium aprine* L. (Rubiaceae) commonly known as clivers, cleavers, bedstraw, catchweed, or goosegrass, is an annual herb with fibrous roots, scrambling-erect stem, dull, yellowish-green leaves, and small white flowers with 4–8 brackets [1]. Cleavers is widely distributed around region of Europe, Northern Africa, and Asia from Great Britain and the Canary Islands to Japan, and it grows in mostly partial shade but can also withstand sunny spells. In general, it thrives in any well-drained soil [2]. Extracts from aerial parts of cleavers are a herbal remedy with monography in British Herbal Pharmacopeia [3]. It is used in the form of infusions by patients as a diuretic, alterative, anti-inflammatory, tonic, and astringent [4]. In folk medicine, cleavers was used topically in Europe, Asia, and the Americas to treat skin diseases [5,6,7,8]. There are also several reports on the application of cleavers preparation in the prevention and treatment of various animal aliments [9,10]. On the European market, there are several drugs and food supplements containing cleavers extracts. Many of them were listed in Martindale Reference [11]. In several remedies, cleavers is listed as an immunomodulatory active herb influencing the defense response of human body. Some drugs containing cleavers are recommended as stimulators of non-specific defense mechanism of the body and as detoxicant [12]. Previous studies on phytochemical composition of aerial parts of plant from *Galium* species showed that they contain as major compounds iridoids (mainly monotropein, asperulosidic acid, and asperuloside), flavonoids (quercetin and kaempferol glycosides) and chlorogenic acids [13,14,15,16]. Also some steroids and alkaloids were found in few studies in *Galium* plants [17]. Our previous research on ethanolic extracts from cleavers indicated that they have some immunostimulatory activity. It was also shown that ethanolic extract consist of mainly chlorogenic acid derivatives, flavonoids and some iridoids [18].

Immunomodulatory effects were discovered in different classes of biologically active compounds, in particular polysaccharides [19]. Indeed, chamomile and marshmallow polysaccharides are active ingredients in the Imupret composite mixture herbal product, which stimulate a non-specific immune response via the enhancement of the phagocytic activity of macrophages and granulocytes.

Leucocytes are the first line of defense of the organism toward skin injuries and infections [20]. They are responsible for the generation of the inflammatory response against pathogens. One of the major forms of the activation of lymphocytes by external factors, i.e., lipopolysaccharide (LPS) is the production/release of significant amounts of reactive oxygen species (ROS). ROS cause bacteria destruction but also may affect healthy tissues by the induction of cell death pathways which is not beneficial for wound healing process in vivo. One of the strategies for the augmentation of the healing process is topical application of remedies that have the antioxidant activity [21].

The aim of this study was to investigate the immunostimulatory activity and antioxidant potential in vitro of raw infusion of cleavers and bioactive fractions namely the polysaccharide complex (PSC), polyphenolic complex (PPC) and pectin (PC). The functional activity of lymphocytes in the reaction of the lymphocyte blast transformation (RLBT) method was used for immunomodulatory activity assays and the direct scavenging of DPPH, NO, and H_2_O_2_ was carried out for the examination of the antioxidant activity.

## 2. Results

### 2.1. Phytochemical Screening of G. aparine Herb Aqueous Extract

The phytochemical screening of the *G. aparine* herb aqueous extract revealed the presence of polysaccharides, flavonoids (flavonols and flavones) and derivatives of phenolic acids, with the results obtained corresponding with previous studies [13,14,15,16,17,18].

### 2.2. Quantification of Main Groups of BAFs

The content of main groups of BAFs in *G. aparine* herb aqueous extract is presented in Table 1 below.

In quantifying main groups of phytochemicals, it was established that the raw extract contained 9.63% (96.3 μg/mg) polysaccharides, 1.87% (18.7 μg/mg) hydroxycinnamic derivates expressed as chlorogenic acid, 0.26% (2.6 μg/mg) flavonoids expressed as rutin, and 1.33% (13.3 μg/mg) polyphenols/tannins expressed as gallic acid. The PPC fraction after the removal of polysaccharides and pectines and contained 2.86% (28.6 μg/mg) hydroxycinnamic derivates expressed as chlorogenic acid, 0.36% (3.6 μg/mg) flavonoids expressed as rutin, and 1.92% (19.2 μg/mg) polyphenols/tannins expressed as gallic acid.

The obtained results proved that raw extract is a rich source of phenolics, especially hydroxycinnamic acid derivatives. The separation of the raw extract in two major fractions (PPC and PSC) allowed the investigation of the bioactivity of each group of compounds using chosen models. This approach could show which phytochemicals contribute to the overall activity of the *G. aparine* extract in the context of its traditional use. The production of PC from the residue, which remains after obtaining the infusion, and the study of its activity, suggests that the complex processing of the *G. aparine* herb is expedient. The quantification of predominant compounds in raw extract and PPC fraction was performed with UHPLC-DAD-MS methodology.

### 2.3. Chemical Composition of Investigated Infusion and BAFs

**Figure 1:**
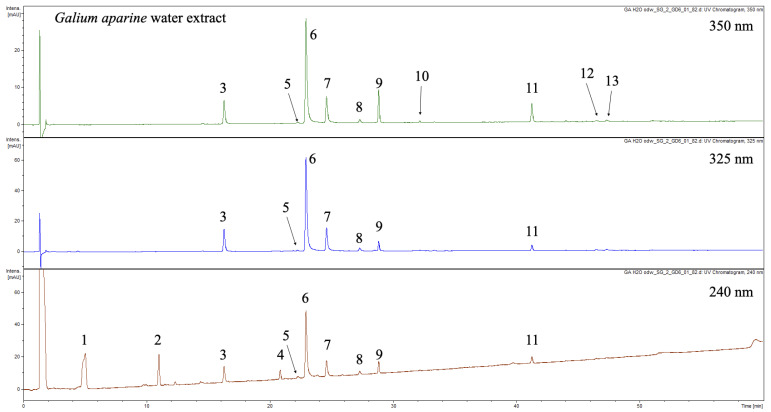
Representative UHPLC-DAD-MS^3^ chromatogram at 350, 325 and 240 nm.

The UHPLC-DAD-MS^3^ analysis revealed that raw extract and PPC fraction contains thirteen major compounds (**1**–**13**, Figure 1, Table 2). Detected compounds were characterized based on UV-Vis spectra and MS spectra. The composition was almost the same as for ethanolic extracts analyzed in our previous research [18]. In brief, three major iridoids namely monotropein (**1**), desacetylasperulosidic acid (**2**) and asperulosidic acid (**4**) were identified. Additionally, seven chlorogenic acids were detected and characterized (**3**, **5**–**8**, **12,** and **13**). The careful analysis of MS spectra and comparison with surrogate standards according to Clifford showed that **3**, **5**–**8**, **12**, and **13** were identified as: 3-*O*-trans-caffeoylquinic acid, 3-*O*-cis-caffeoylquinic acid, 5-*O*-trans-caffeoylquinic acid, 4-*O*-trans-caffeoylquinic acid, 5-*O*-cis-caffeoylquinic acid, 3,4-*O*-trans-dicaffeoylquinic acid, and 3,5-*O*-trans-dicaffeoylquinic acid respectively [22]. Apart from iridoids and chlorogenic acids, the extract contained flavonoids (**9**–**11**). Compounds **9** and **11** were identified as quercetin 3-*O*-rhamnoglucoside-7-*O*-glucoside and rutin, respectively. Both compounds were previously detected in ethanolic extracts from *G. aparine*. Compound **10** was identified as an analogue of **9** with kaempferol as a core aglycone. Due to the similarities of **9** and **10**, compound **10** was tentatively identified as kaempferol *O*-rhamnodihexoside, most probably 3-*O*-rhamnoglucoside-7-*O*-glucoside. This compound was detected in *G. aparine* for the first time and was not reported in any other *Galium* species. In our previous study it was not detected due to low abundance in analyzed extracts. In order to full confirm the chemical structure of **10** isolation and spectral analysis is needed.

Using the developed UHPLC-DAD method, twelve out of thirteen detected compounds were quantified. Phenolic were present only in raw extract and PPC fraction. The analysis of PC and PSC complexes showed that no phenolics are present (data not shown). The quantification revealed that raw aqueous extract contains significant amounts of iridoids mainly monotropein (**1**) and deacetylasperulosidic acid (**2**) (5.27 and 1.96 µg/mg, respectively). The third compound (**4**) from this class of phytochemicals was present at the concentration of 0.524 µg/mg. Chlorogenic acids were the second group of natural products occurring in the analyzed samples in high quantities. The caffeoylquinic acids-complex consisted mainly chlorogenic acid (**6**, 4.68 µg/mg), neochlorogenic acid (**3**, 0.966 µg/mg), and cryptochlorogenic acid (**7**, 1.140 µg/mg). Dicaffeoylquinic acid isomers (**12** and **13**) were present in amount below 0.1 µg/mg in analyzed samples. Apart from trans isomers of chlorogenic acids, cis derivatives were also detected and quantified. However, their presence may be connected with the extraction procedure and they should be considered as artefacts [22]. The major flavonoid present in analyzed samples was compound **9** which was present at the concentration of 0.96 µg/mg. Rutin (**11**) was present at the concentration of 0.064 µg/mg. Compound **10** was not quantified due to low abundance.

The analysis of PPC fraction showed that it contains same phenolics as raw aqueous extract. Apart from phenolics the extraction procedure caused the presence of iridoids. All compounds were also quantified. The processing of the extract caused increase in the concentration of phenolic compounds and iridoids. The content of iridoids varied from 0.806 to 8.120 µg/mg, the amount of chlorogenic acids was between 0.121 and 7.124 µg/mg. Flavonoids in the PPC fraction were also quantified and the content was 0.140 µg/mg for **9** and 0.98 µg/mg for rutin (**11**).

To compare results obtained for non-specific chemical methods sums of each group of phytochemicals was calculated from UHPLC quantification. The sum of caffeoylquinic acid derivatives was 7.244 µg/mg for raw extract and 10.965 µg/mg for PPC. The amount of flavonoids quantified with UHPLC was 0.160 µg/mg for raw extract and 0.238 µg/mg for PPC fraction. Finally, iridoids in aqueous extracts were quantified as 7.753 µg/mg and 11.990 µg/mg for PPC fraction. The quantification with chemical approach showed higher amount that is obvious taking into account fact that those methods are non-specific and other group of compounds may interfere in the quantification process. Iridoids were not quantified with the chemical method because their content was not expected to be so high in the analyzed material.

### 2.4. In Vitro Reaction of Lymphocyte Blast Transformation

The research presented is the first known study of the immunomodulatory activity of the *G. aparine* herb aqueous extract, PPC, PSC, and PC complex.

It was established that all studied samples considerably stimulate the transformational activity of peripheral blood mononuclear cells. Under the influence of the substances under study, 36.3–65.5% of mononuclear cells became involved in the proliferation process, which indicates the stimulating effect of the substances on T- and B-lymphocytes, which is higher than that of the reference substance PHA with the exception of PPC (Table 3).

The summary of the results the lymphocyte blast transformation model is presented in Table 3. The obtained data show that all substances under study from *G. aparine* have an immunomodulatory potential.

The highest immunostimulatory activity was observed for the aqueous extract at the concentration of 250 µg/mL which was 1.36 times as high as that of the reference substance PHA. Surprisingly, lower stimulation of lymphocytes was established at a concentration of 500 µg/mL. However, the observed activity was still significantly higher than for positive control PHA.

Furthermore, it was observed that aqueous extract from *G. aparine* displays higher activity than ethanolic preparations investigated previously [18]. However, the removal of polysaccharides from the aqueous extract results in a significant decrease in the immunomodulatory activity. The performed experiments the PPC showed lower activity than the reference substance PHA, comparable to that of ethanolic extracts [13].

The level of the immunomodulatory activity established for PSC was higher than that of PHA, but insignificantly lower than that of the aqueous extract. The obtained results indicated that the polysaccharides occurring in the aqueous extract from *G. aparine* herb contribute to the overall activity of the extract more than phenolics. It was shown that the removal of the polysaccharides causes impairment of immunostimulatory activity in the investigated model.

Comparing the activity established for PSC and PC complexes, it was found that the activity of the PC was insignificantly lower than that of the PSC (Table 3).

### 2.5. Antioxidant Activity of Infusion and BAFs Using Cell Free Models

**Figure 2:**
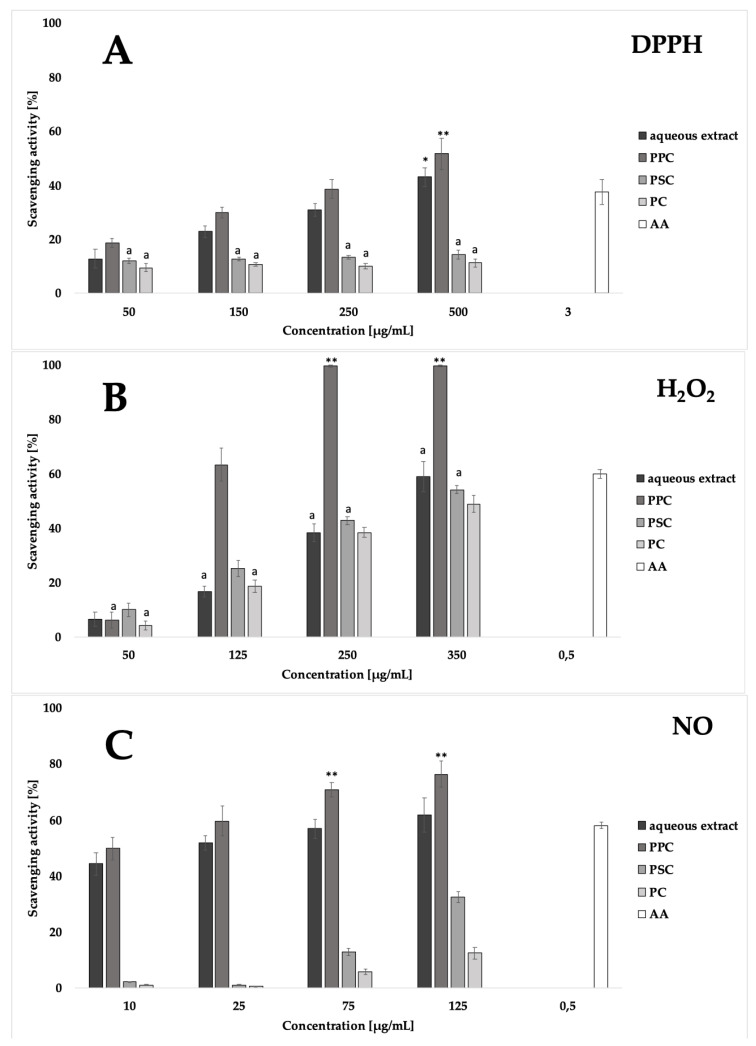
Scavenging activity of raw extract and BAFs against DPPH (**A**), H_2_O_2_ (**B**) and NO (**C**) species, AA—ascorbic acid (positive control). *, **—significant stronger activity in comparison to the control at *p* < 0.05 and *p* < 0.01, respectively, a—not significant difference between the tested extracts activity at *p* < 0.05.

The anti-scavenging activity of the raw extract and its fractions was established in cell-free models. The scavenging potency of analyzed samples against DPPH, H_2_O_2_, and NO species was established (Figure 2). Concentrations of extract and fractions were chosen so the dose-dependent effect can be observed. The weakest activity was observed in the case of DPPH scavenging ability. Aqueous extract at the highest used concentration of 500 µg/mg has the activity comparable to the positive control. This is a considerable weak result. Raw extract and PPC fraction showed similar activity, while PC and PSC fractions had poor scavenging effect. This observation is in the agreement with the fact that they did not contain any phenolics or iridoids that show antioxidant potential. In the case of scavenging activity of H_2_O_2_ it was showed that the strongest activity has PPC fraction. It was significantly better antioxidant at the concentration of 125 µg/mg compare to ascorbic acid at 500 µg/mg used as a positive control. Aqueous extract showed significantly weaker activity (Figure 2). Surprisingly in the case of H_2_O_2_-scavenging raw extract acted in the similar manner as phenolics and iridoids free PC and PSC fractions (Figure 2). In this method the enzymatic reaction is used, and compounds contained in PC/PSC fraction may interfere in the assay. It was suspected that PC and PSC complexes will show poor activity compared to the extract, but this was not the case. It is not clear what is the mechanism of this effect. The strongest antioxidant potential was observed in the NO scavenging assays. Fractions and the extract were tested in the concentration rage from 10 to 125 µg/mg. Complexes PC and PSC showed almost no activity. the scavenging power of aqueous extract and PPC fraction was comparable in the whole concentration range, however it was significantly stronger than observed for the positive control (Figure 2).

## 3. Discussion

In the traditional medicine *G. aparine* is used as topical agent for skin infections and for wounds [2,8]. Obtained results show that it has stimulatory activity in the lymphocyte blast assays. The extract significantly activates the host dependent defense response. This is crucial in the case of bacterial infections. It was shown previously that the immunostimulatory activity of several medicinal plant materials has beneficial effect in the treatment of skin diseases [24]. In the literature the immunostimulatory activity of plant extracts is often attributed to the presence of high molecular polysaccharides [24,25,26]. This was also the major hypothesis explaining the stimulatory activity of our previous study on ethanolic extracts from *G. aparine* [18]. The current evaluation of different complexes obtained from the raw extract partially confirmed that PSC and PC significantly contribute to immunostimulatory activity of raw extract. The values obtained in the RLBT assays were similar for PSC and PC fractions as for the raw infusion (Table 3). This suggests that these two groups of natural products are responsible for the immunostimulatory effect of whole infusion. However, it also turned out that PPC fraction containing a high amount of phenolics and iridoids was active in the RLBT assay. The observed effect was though significantly weaker than for PSC, PC and raw extract. Nevertheless, it can be assumed that all classes of phytochemicals act synergistically in *G. aparine* preparations used by the patients. In antioxidant assays, it was shown that the raw water extract has strong scavenging activity in the investigated models. It also was proved that mainly PPC fraction contribute to this activity. The scavenging activity of *G aparine* extract can be considered as beneficial in the case of topical application. The overstimulated immune system causes a massive production of ROS which can cause tremendous damage not only to bacteria, but also to healthy tissues of the host. On the other hand, the stimulation of lymphocytes leads to the increased production of both ROS and cytokines. The plant extract exhibiting antioxidant activity can easily scavenge ROS protecting tissues from damage. At the same time several studies suggest that increased levels of cytokines like TNF-alpha or IL-8 can promote and enhance wound healing [2]. So, the observed effects of *Galium* infusion can be considered to be of importance for topical application on injuries through the stimulation immune system to eliminate pathogens using other mechanisms, such as cytokine production, phagocytosis and others.

## 4. Materials and Methods

### 4.1. Plant Material

Cleavers (*G. aparine*) herb was collected from cultivation at the Botanical garden of the National University of Pharmacy, Kharkiv, Ukraine in May, 2017. Voucher specimens no 20052017–23052017 were deposited at the Department of Pharmacognosy (National University of Pharmacy, Kharkiv, Ukraine). The identity of the plant was established with the consulting assistance of T. Gontova, D.Sc. [27].

### 4.2. Chemicals

Ethanol, hydrochloric acid, acetic acid, lead (II) acetate, aluminum chloride, granulated zinc, ferric (III) chloride, and sodium nitroprusside (SNP) were purchased from Avantor (Gliwice, Poland). Gallic acid, chlorogenic acid and rutin were purchased from Merck, DE. Ascorbic acid, 2,2-diphenyl-1-picrylhydrazyl (DPPH) radical, horseradish peroxidase (HRP), hydrogen peroxide (H_2_O_2_), loganin, isoquercitrin and diaminofluorescein (DAF) were purchased from Sigma-Aldrich, St. Louis, MO, USA. Phosphate-buffered saline (PBS) was purchased from Biomed (Lublin, Poland). Water was obtained using Milli-Q Plus, MILLIPORE (Billerica, MA, USA) (18.2 MΩ cm).

### 4.3. Preparation of the Extract and BAF Complexes

Prior to the extraction, *G. aparine* herb was crushed to a particle size 3–4 mm. The extraction was carried out at a general ratio of the plant material: solvent of 1:10 on heating with reflux, with water used as a solvent. The extraction was repeated three times under the same conditions (30 min each). The obtained extracts were combined, concentrated on a vacuum rotary evaporator to dryness.

At the same time, in the same conditions, the extract was obtained from which polysaccharides were precipitated with three volumes of 96% ethanol, separated by centrifugation (10 min, 3000 rpm), re-washed with 96% ethanol, and centrifuged under the same conditions. The resulting polysaccharide complex was dried to an air-dry state (PSC). The filtrate residue after the PSC precipitation was concentrated to dryness to obtain polyphenolic complex (PPC).

To extract pectins, the accurately weighed residue after the aqueous extraction of the species under study was acidified with 0.33% solution of oxalic acid at the ratio of the plant material: solvent of 1:7.5 and heated on a boiling water bath for 1 h (solution of pH 3). Then, the purified water was added to achieve the ratio of plant material: solvent of 1:20, and a 25% solution of ammonium hydroxide was added dropwise until pH 6, and the mixture was heated on a boiling water bath for 1 h. The extract was filtered, evaporated on the vacuum rotary evaporator down to one-third of its volume and the pectins was precipitated with three volumes of 96% ethanol. The precipitate was separated by centrifugation (10 min, 3000 rpm), re-washed with 96% ethanol, re-centrifuged, and dried. The extraction with purified water was repeated three times at the ratio of plant material: solvent of 1:20. The precipitated pectins (PC) were dried and weighed.

### 4.4. Preliminary Phytochemical Screening of G. aparine Herb Extract and PPC

The preliminary phytochemical screening was performed with the use of generally accepted methods and techniques of phytochemical analysis as described earlier [18,28].

### 4.5. Quantification Phytochemicals by Using Chemical Methods

Chemical methods were used for the estimation of total polysaccharides, total flavonoids, total polyphenols, and total hydroxycinnamic acid derivatives contents. Assays were performed for raw infusion and BAFs. All samples were repeated three times. Total flavonoids were tested using Christ-Muller methodology (aluminum chloride assay, quercetin 3-*O*-rhamnoglucoside as a standard was used) according to European Pharmacopeia [29]. Caffeic acid derivatives were quantified spectrophotometrically according to Yezerska et al. [30]. Total polyphenols was established using methodology published by Kovalyova et al. [31]. Total polysaccharides and pectins were tested gravimetrically according to Pharmacopeias [29,32].

### 4.6. UHPLC-DAD-MS^3^ Analysis of Galium Aparine Infusion and Bioactive Fractions (BAFs)

The UHPLC analysis was performed using Ultimate 3000 series system (Dionex, Idstein, Germany) equipped with dual low-pressure gradient pump with vacuum degasser, an autosampler, a column compartment, and a diode array detector coupled with Amazon SL ion trap mass spectrometer (Bruker Daltonik GmbH, Bremen, Germany). The separation of compounds in the analyzed extract was carried out with Kinetex XB-C_18_ analytical column (100 × 2.1 × 1.9 µm), Phenomenex (Torrance, CA, USA). Column temperature was maintained at 25 °C. Elution was conducted using mobile phase A (0.1% HCOOH in deionized water) and mobile phase B (0.1% HCOOH in acetonitrile) with linear gradient as follows: 0 min 1%B, 60 min 26%B. The flow rate was set to 0.300 mL/min. Five µL of each sample was introduced to the column by the autosampler. UV–vis spectra were recorded in the range of 190–450 nm. Chromatograms were acquired at 240, 325 and 350 nm. The eluate was introduced directly into mass spectrometer. The ion trap mass spectrometer was equipped with ESI interface. The parameters for ESI source were set as follows: nebulizer pressure 40 psi; dry gas flow 9 L/min; dry temperature 300 °C; and capillary voltage 4.5 kV. Analysis was carried out using scan from *m*/*z* 70–2200. Compounds were analyzed in negative ion mode. The MS^2^ and MS^3^ fragmentations were performed using smart frag mode. Compounds were identified according to the signals obtained from DAD and MS detectors after the comparison with chemical standards available and proper literature [23,33].

### 4.7. Quantification of Phenolics and Iridoids Contained in Analyzed Samples

Accurately weighted around 10 mg of each sample was dissolved in DMSO to obtain solution at the concentration of 10 mg/mL. Solutions were filtered through 0.45 μm PVDF syringe filers and subjected to UHLPC-DAD analysis. Stock solutions of standards for calibration (chlorogenic acid, loganin and isoquercitrin) were prepared by dissolving accurately weighted 1 mg of standard in 1 mL of methanol. Next 50 μL of each solution was mixed with 850 μL of 0.1% formic acid in water to obtain final concentration of 50 μg/mL of each compound. Calibration curves were plotted as peak area vs. amount of the compound injected to UHPLC column. The linear range of calibration curves for each compound was 50–800 ng per injection. Regression factors for chlorogenic acid, loganin and isoquercitrin were higher than 0.9980. Phenolic acids detected in samples were quantified as chlorogenic acid equivalents, iridoids as loganin equivalents, and flavonoids as isoquercitrin equivalents.

### 4.8. Study of Immunomodulatory Activity

The stimulatory of lymphocytes was established according to the previously published methodology [18]. In brief, PBMC fraction was isolated by the centrifugation in Ficoll gradient. The blood was collected from healthy volunteers after meeting all criteria required by WMA declaration of Helsinki. Cells were with tested extract and fractions in comparison to the positive control PHA.

### 4.9. Evaluation of Antioxidant Activity of Infusion and BAFs Using Cell Free Models

#### 4.9.1. Scavenging of the DPPH Radical

Scavenging of the DPPH (2,2-diphenyl-1-picrylhydrazyl) radical was examined using the method of Choi [34]. One hundred µL of extract solutions in 50% (*v*/*v*) ethanol, at concentrations of 50, 150, 250, and 500 µg/mL were mixed in a 96-well plate with 100 µL of a 0.02 mM solution of the DPPH radical dissolved in 96% (*v*/*v*) ethanol. After 30 min of incubation in the dark at room temperature, absorbance at 518 nm was measured in a Synergy 4 microplate reader. The scavenging rate of the DPPH radical was calculated relative to a control without the tested extracts. Ascorbic acid was used as a positive control.

#### 4.9.2. Scavenging of Hydrogen Peroxide

Scavenging of hydrogen peroxide (H_2_O_2_) was performed with horseradish peroxidase as described by O’Dowd [35]. Then, 50 µL of the extract in PBS at concentrations of 50, 125, 250, and 350 µg/mL was mixed in a white 96-well plate with 50 µL of horseradish peroxidase (solution in PBS, prepared ex tempore, 98.8 mU HRP), 50 µL hydrogen peroxide (solution in PBS, prepared *ex tempore*, 0.00075% H_2_O_2_) and 50 µL of luminol (0.005 mg/mL in PBS). The chemiluminescence was measured at room temperature in the absence of light, 7.5 min after the addition of the luminol solution. The reader was set to read luminescence at sensitivity 75. The percent of inhibition of the HRP/hydrogen peroxide system was calculated in comparison to the control without test extracts. Ascorbic acid was used as a positive control.

#### 4.9.3. Scavenging of Nitrogen Oxide

Fifty µL of extracts solution at concentration of 10, 25, 75 and 125 µg/mL was mixed with 50 µL of 5 mM DAF-2, 50 µL of 3 mg/mL SNP, and 50 µL of PBS. Measurements were made after 20 min at 37 °C in the dark on a black 96-well plate in a microplate reader at excitation and emission wavelengths of 495 and 515 nm, respectively. The scavenging of nitrogen oxide was calculated relative to controls without tested extracts. Ascorbic acid was used as a positive control.

### 4.10. Statistical Analysis

All statistical analyses were carried out in accordance with the requirements of the State Pharmacopoeia of Ukraine using Microsoft Office [32]. Differences between groups were statistically analyzed using one-way analysis of variance (ANOVA). The results were expressed as mean ± standard deviation (SD). *p* values less than 0.05 or 0.001 were considered statistically significant.

## 5. Conclusions

The first-obtained aqueous extract, polysaccharides, and pectin from *G. aparine* herb were phytochemically profiled and evaluated for potential immunomodulatory and scavenging activities. All substances from *G. aparine* herb significantly stimulate the transformational activity of immunocompetent blood cells, with the aqueous extract being the most active. All samples also displayed antioxidant properties against some ROS used in the assays. Obtained data at least partly justify the traditional use of *G. aparine* as a topical remedy for skin infections and for wounds. However, further research involving human skin cells and the interaction of lymphocytes with them as well as animal studies are needed for the full confirmation of this thesis.

## Figures and Tables

**Table 1:** The content of main groups of phytochemicals in *G. aparine* herb aqueous extract.

Substances	Extraction Yield (mg/g)	Content (μg/mg)			
		Polysaccharides	Hydroxycinnamic Derivates	Flavonoids	Polyphenols
Aqueous extract	283.9 ± 14.1	96.3 ± 4.8	18.7 ± 0.9 a	2.6 ± 0.1 a	13.3 ± 0.6 a
PPC	187.6 ± 14.1	-	28.6 ± 0.6 b	3.6 ± 0.1 b	19.2 ± 0.5 b

Note: PPC = polyphenolic complex, different letters a, b—show statistical significance *p* < 0.05.

**Table 2:** UHPLC-DAD-MS and quantification data of aqueous extract and PPC fraction.

No	Identification	Retention Time (min)	UV-Vis Maxima (nm)	MS^−^ Ions	MS^2−^ Ions	MS^3−^ Ions	Content in RAW Extract (µg/mg)	Content in PCC Fraction (µg/mg)
1	monotropein ^t^	4.8	238	389	**227**, 209b, 191, 165, 147, 137	183, 165, 153b, 137	5.270 ± 0.160	8.120 ± 0.112
2	desacetyloasperulosidic acid ^t^	11.0	239	389	371, **227b**, 209, 191, 183, 165, 139, 119	183b, 165	1.960 ± 0.030	3.064 ± 0.064
3	3-*O*-*trans*-caffeoylquinic acid ^c^	16.3	300sh, 324	353	191b, 179, 173, 135	-	0.966 ± 0.016	1.384 ± 0.098
4	asperulosidic acid ^t^	20.9	237	431	371, **269**, 251b, 225, 165	225, 165b	0.524 ± 0.006	0.806 ± 0.021
5	3-*O*-*cis*-caffeoylquinic acid ^c^	22.4	300sh, 324	353	191b, 179, 173	-	0.080 ± 0.001	0.121 ± 0.004
6	5-*O*-*trans*-caffeoylquinic acid ^c^	23.0	301sh, 324	353	**191b**, 179, 164	179b, 164	4.680 ± 0.080	7.124 ± 0.112
7	4-*O*-*trans*-caffeoylquinic acid ^c^	24.8	300sh, 325	353	**191**, 179, 173b	179, 173b	1.140 ± 0.020	1.754 ± 0.062
8	5-*O*-*cis*-caffeoylquinic acid ^c^	27.4	299sh, 324	353	191b	-	0.191 ± 0.019	0.289 ± 0.011
9	quercetin 3-*O*-rhamnoglucoside-7-*O*-glucoside ^s^	29.1	260, 352	771	**609b**, 301	343, 301b, 271, 255, 179	0.096 ± 0.003	0.140 ± 0.002
10	kaempferol *O*-rhamnodihexoside ^t^	32.5	261, 342	755	**593b**, 285	533, 285b, 267, 257, 240	n.q.	n.q.
11	quercetin 3-*O*-rhamnoglucoside^s^ (rutin) ^s^	41.4	261, 353	609	465, 343, 301b	343, 301b, 255	0.064 ± 0.002	0.098 ± 0.007
12	3,4-*O*-*trans*-dicaffeoylquinic acid ^c^	46.4	301sh, 325	515	**353b**, 335, 299, 255, 203, 191, 179, 173, 135	191, 179, 173b, 135	0.095 ± 0.004	0.143 ± 0.009
13	3,5-*O*-*trans*-dicaffeoylquinic acid ^s^	47.4	300sh, 324	515	353b	191b, 179, 173	0.092 ± 0.006	0.150 ± 0.011

^t^—tentative assignment based on literature reports on *Galium* species, ^c^—assignment according to Clifford et al. [22,23], ^s^—comparison with chemical standard was made, n.q.—not quantified, in bold—ion subjected to MS^3^ fragmentation.

**Table 3:** The effect of aqueous extract and other substances from *G.* *aparine* on the indices of lymphocyte blast transformation (X ± m), *n* = 5.

Extract	Extract Concentration (μg/mL)	RLBT, %
Aqueous extract	150	60.8 ± 3.2 *
	250	65.5 ± 3.3 *
	500	61.6 ± 3.5 *
PPC	150	37.6 ± 2.6 *
	250	39.8 ± 2.6 *
	500	36.3 ± 2.3 *
PSC	150	48.7 ± 3.5 ^#^
	250	58.6 ± 3.2 *
	500	55.7 ± 3.3 *
PC	150	39.0 ± 3.2 *
	250	52.7 ± 4.1 *
	500	59.3 ± 3.1 *
PHA	250	48.1 ± 2.1 ^#^
Spontaneous RLBT	-	8.5 ± 0.7

Note: PPC = polyphenolic complex; PSC = polysaccharide complex; PC = pectin; PHA = phytohemagglutinin; RLBT = the reaction of lymphocyte blast transformation; *, ^#^—*p* < 0.05 in comparison with PHA, different markers indicate statistically significant differences.
